# Identifying Resistance Mechanisms against Five Tyrosine Kinase Inhibitors Targeting the ERBB/RAS Pathway in 45 Cancer Cell Lines

**DOI:** 10.1371/journal.pone.0059503

**Published:** 2013-03-29

**Authors:** Zsófia Pénzváltó, Bálint Tegze, A. Marcell Szász, Zsófia Sztupinszki, István Likó, Attila Szendrői, Reinhold Schäfer, Balázs Győrffy

**Affiliations:** 1 1st Department of Pediatrics, Semmelweis University, Budapest, Hungary; 2 2nd Department of Pathology, Semmelweis University, Budapest, Hungary; 3 Gedeon Richter Plc., Budapest, Hungary; 4 Department of Urology, Semmelweis University, Budapest, Hungary; 5 Laboratory of Functional Genomics and of Molecular Tumor Pathology, Charité Universitätsmedizin, Berlin, Germany; 6 Research Laboratory of Pediatrics and Nephrology, Hungarian Academy of Sciences, Budapest, Hungary; Universität Heidelberg, Germany

## Abstract

Because of the low overall response rates of 10–47% to targeted cancer therapeutics, there is an increasing need for predictive biomarkers. We aimed to identify genes predicting response to five already approved tyrosine kinase inhibitors. We tested 45 cancer cell lines for sensitivity to sunitinib, erlotinib, lapatinib, sorafenib and gefitinib at the clinically administered doses. A resistance matrix was determined, and gene expression profiles of the subsets of resistant vs. sensitive cell lines were compared. Triplicate gene expression signatures were obtained from the caArray project. Significance analysis of microarrays and rank products were applied for feature selection. Ninety-five genes were also measured by RT-PCR. In case of four sunitinib resistance associated genes, the results were validated in clinical samples by immunohistochemistry. A list of 63 top genes associated with resistance against the five tyrosine kinase inhibitors was identified. Quantitative RT-PCR analysis confirmed 45 of 63 genes identified by microarray analysis. Only two genes (*ANXA3* and *RAB25*) were related to sensitivity against more than three inhibitors. The immunohistochemical analysis of sunitinib-treated metastatic renal cell carcinomas confirmed the correlation between RAB17, LGALS8, and EPCAM and overall survival. In summary, we determined predictive biomarkers for five tyrosine kinase inhibitors, and validated sunitinib resistance biomarkers by immunohistochemistry in an independent patient cohort.

## Introduction

Targeted therapy in cancer treatment refers to the application of special agents acting on specific molecular features of signal transduction pathways involved in the development of the cancerous phenotype. Erlotinib, gefitinib, sorafenib, sunitinib and lapatinib are all clinically used tyrosine kinase inhibitors (TKIs) targeting receptors and downstream members of the ERBB/RAS pathway [1]. Erlotinib and gefitinib are reversible epidermal growth factor receptor (EGFR) tyrosine kinase inhibitors used in the treatment of non-small cell lung cancer. About 10% of the patients respond to the treatment in the European and Northern American population [2]. Lapatinib inhibits the tyrosine kinase domain of the epidermal growth factor receptor (EGFR) and human epidermal growth factor receptor 2 (HER2). It is approved for the treatment of breast cancer, where the overall response rate to this treatment is 24% [3]. Sorafenib inhibits RAF, VEGFR, PDGFR, Flt-3, c-Kit receptors. The partial response rate is 10%, when it is administered for patients with advanced renal-cell carcinoma [4]. Sunitinib is a small-molecule multi-targeted receptor tyrosine kinase (RTK) inhibitor that was approved by the FDA for the treatment of renal cell carcinoma (RCC) and imatinib-resistant gastrointestinal stromal tumor (GIST). Objective response rate is 31% in the first line treatment of renal cell carcinoma [5]. Because of the low overall response rates of 10–47% [6]–[10], there is an increasing need for biomarkers predicting response to targeted therapy treatment.

Besides pharmacokinetic parameters, a tumor can deploy different molecular mechanisms to achieve resistance against targeted therapy agents: the target molecule may be subject to modification, downstream alterations of the pathway may lead to resistance against an agent targeting an upstream molecule, or other pathways may be activated which alternatively mediate cancer cell survival and proliferation. For example, the T790M mutation of the *EGFR* gene retains the ability of the receptor to activate the downstream pathway but simultaneously decreases binding of gefitinib and erlotinib to the receptor and thus leads to drug resistance [11]. *MET* amplification causes resistance against erlotinib and gefitinib through the activation of alternative pathways [12]. Interleukine-8 can activate an alternative pathway leading to sunitinib resistance [13]. Mutations of the genes of downstream members of the pathway can also contribute to resistance against targeted therapy agents, as described before in case of *KRAS* [14], *PTEN* [15], *BRAF* [16], and *PIK3CA* [17]. When a downstream component of the signaling system activates the pathway, inhibition by the blockade of an upstream member was shown to be ineffective. These downstream changes can be used as negative predictors for agents acting upstream of this addictive element of the pathway, as described before for *KRAS* [18]. If *KRAS* harbors an activating mutation, agents acting on EGFR will not have any effect on tumor growth [19].

Previous studies have already described that the use of gene expression data, coupled with *in vitro* drug sensitivity assays, can be used to develop signatures that could classify response to conventional anticancer agents [20], [21]. In another study, a panel of cancer cell lines was treated with dasatinib, a multitarget kinase inhibitor, and sensitivity to the drug was measured. In parallel, expression data generated from the same panel of cell lines was used to develop a signature to predict sensitivity to the drug [22]. In a different study, a panel of lung cancer cell lines was used to develop gene expression signatures that predict sensitivity to the EGFR inhibitors gefitnib [23] and erlotinib [24]. Finally, the common significant genes of an *in vitro* and an *in vivo* study were able to predict response to rapamycin [25]. Although focused on single therapeutic agents in one type of cancer, these studies already demonstrated the power of gene expression profiles to predict response to a specific agent.

In this present study, we took a broader approach aiming to identify gene signatures associated with intrinsic resistance against 5 already approved tyrosine kinase inhibitors targeting the ERBB/RAS-pathway. To obtain new predictive biomarkers, we correlated the sensitivity of 45 cell lines representing 15 different cancer entities to expression patterns. The best performing candidate genes were then validated using qRT-PCR. Finally, clinical validation was performed using immunohistochemistry based on tissue microarrays on a set of renal cell carcinomas from patients treated with sunitinib.

## Materials and Methods

### Ethics Statement

The approval number for the sample collection by the National Scientific and Research Ethics Committee (ETT-TUKEB) (Hungary) is #185/2007. General informed consent was obtained before the surgery. The National Scientific and Research Ethics Committee did not request a specific written permission, because, it was a retrospective study, and the patients were handled anonymously.

### Cell Culture

**Figure 1:**
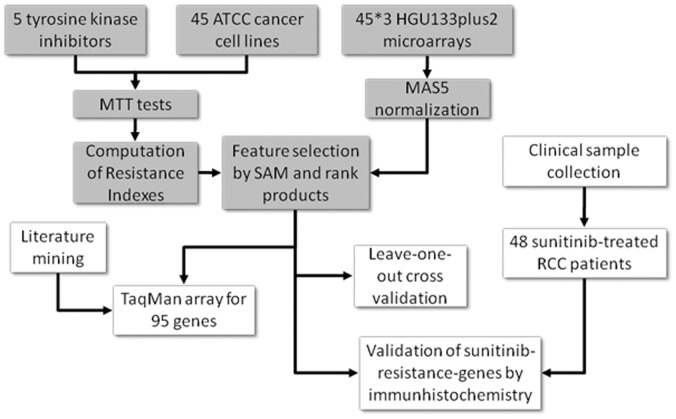
Overview of the study. Boxes with grey background represent training steps, while white background represents validation steps.

We obtained 45 ATCC cell lines. Before selection, the absence of *KRAS* mutation in the cell lines was confirmed using the Catalogue of Somatic Mutations in Cancer (search done on the 25^th^ of June 2010). The cells were cultured according to the ATCC protocols (http://www.lgcstandards-atcc.org/). Additionally, antibiotics (Penicillin-streptomycin, Invitrogen, cat. no.: 15070-063, Amphotericin B, Invitrogen, cat. no.: 15290-026) were added. The cell lines are summarized in **Table 1**. An overview of the study is presented in **Figure 1**.

**Table 1:** Resistance characteristics of the 45 cell lines investigated.

Cell line	Origin	ATCC	lapatinib		erlotinib		sorafenib		sunitinib		gefitinib	
CCRF-CEM	ALL	CCL-119	**SW-948**	**0,26**	**NCI-H441**	**−0,11**	**NCI-H69**	**−1,53**	**A-375**	**−0,15**	**Hep-3B**	**−0,09**
MOLT-4	ALL	CRL-1582	**K-562**	**0,43**	**HCT-15**	**−0,08**	**NCI-H441**	**−1,25**	**CAOV3**	**0,07**	**HCT-15**	**0,06**
K-562	Bonemarrow	CCL-243	**NCI-H358**	**0,48**	**CAOV3**	**−0,02**	**A-375**	**−1,04**	**HCT-15**	**0,08**	**SW-403**	**0,11**
SK-N-AS	Brain	CRL-2137	**HCT-8**	**0,48**	**SW-948**	**0,09**	**CAOV3**	**−0,99**	**SW-948**	**0,21**	**C-4II**	**0,18**
BT-20	Breast	HTB-19	**HCT-15**	**0,49**	**A-375**	**0,22**	**Hep-3B**	**−0,91**	**NCI-H441**	**0,31**	**NCI-H358**	**0,22**
MCF-7	Breast	HTB-22	**HT-1080**	**0,54**	**NCI-H1993**	**0,24**	**C-4I**	**−0,68**	**NCI-H358**	**0,33**	**NCI-H69**	**0,23**
RAJI	Burkitt’s L	CCL-86	**NCI-H441**	**0,56**	**NCI-H1650**	**0,28**	**SW-620**	**−0,63**	**SW-620**	**0,35**	**WIDR**	**0,28**
C-33A	CervixUteri	HTB-31	**MCF-7**	**0,62**	**NCI-H358**	**0,30**	**HT-1080**	**−0,58**	**SW-480**	**0,38**	**CAOV3**	**0,29**
C-4I	Cervix Uteri	CRL-1594	**CAOV3**	**0,65**	**C-4II**	**0,31**	**HCT-15**	**−0,57**	**MCF-7**	**0,39**	**SNU-449**	**0,29**
C-4II	Cervix Uteri	CRL-1595	**SNU-475**	**0,65**	**SW-480**	**0,39**	**DMS-79**	**−0,53**	**HCT-8**	**0,41**	**HT-1080**	**0,29**
HCT-15	Colon	CCL-225	**Hep-3B**	**0,66**	**NCI-H661**	**0,42**	**C-4II**	**−0,50**	**A-427**	**0,41**	**A-375**	**0,30**
HCT-8	Colon	CCL-244	**NCI-H1650**	**0,71**	**SNU-449**	**0,43**	**NCI-H1993**	**−0,45**	**Hep-3B**	**0,43**	**K-562**	**0,31**
SW-403	Colon	CCL-230	**A-375**	**0,73**	**C-4I**	**0,47**	**SW-480**	**−0,41**	**C-4II**	**0,43**	**HCT-8**	**0,34**
SW-480	Colon	CCL-228	**HOS**	**0,73**	***K-562***	***0,48***	*SHP-77*	**−** *0,36*	**NCI-H1650**	**0,44**	*NCI-H661*	*0,36*
SW-620	Colon	CCL-227	**BT-20**	**0,74**	*SNU-475*	*0,50*	*K-562*	**−** *0,34*	*HT-1080*	*0,51*	*MCF-7*	*0,41*
SW-948	Colon	CCL-237	*SHP-77*	*0,77*	*MCF-7*	*0,51*	*A-427*	**−** *0,33*	*NCI-H82*	*0,51*	*C-4I*	*0,41*
WIDR	Colon	CCL-218	*CCRF-CEM*	*0,78*	*Hep-3B*	*0,51*	*HCT-8*	**−** *0,31*	*ES-2*	*0,51*	*CCRF-CEM*	*0,43*
HT-1080	Fibrosarcoma	CCL-121	*SNU-449*	*0,78*	*CCRF-CEM*	*0,55*	*NCI-H358*	**−** *0,30*	*CCRF-CEM*	*0,51*	*SNU-475*	*0,47*
A-498	Kidney	HTB-44	*C-4II*	*0,78*	*NCI-H69*	*0,55*	*SW-403*	**−** *0,28*	*SNU-475*	*0,51*	*SW-620*	*0,47*
Hep-3B	Liver	HB-8064	*NCI-H1993*	*0,82*	*SW-403*	*0,55*	*SNU-475*	**−** *0,28*	*WIDR*	*0,53*	*NCI-H82*	*0,47*
SNU-182	Liver	CRL-2235	*SW-480*	*0,82*	*HOS*	*0,58*	*COLO-668*	**−** *0,25*	*NCI-H1993*	*0,54*	*SW-480*	*0,48*
SNU-423	Liver	CRL-2238	*C-33A*	*0,87*	*HT-1080*	*0,58*	*CCRF-CEM*	**−** *0,19*	*NCI-H69*	*0,56*	*ES-2*	*0,51*
SNU-449	Liver	CRL-2234	*WIDR*	*0,88*	*C-33A*	*0,59*	*MOLT-4*	**−** *0,18*	*HOS*	*0,59*	*NCI-H1993*	*0,51*
SNU-475	Liver	CRL-2236	*AN3-CA*	*0,89*	*AN3-CA*	*0,60*	*MCF-7*	**−** *0,16*	*SNU-449*	*0,60*	*NCI-H441*	*0,51*
A-427	Lung	HTB-53	*SNU-423*	*0,90*	*ES-2*	*0,61*	*NCI-H1975*	**−** *0,14*	*C-4I*	*0,61*	*C-33A*	*0,53*
COLO-668	Lung	87061209	*A-498*	*0,91*	*SW-620*	*0,61*	*SW-948*	**−** *0,12*	*MOLT-4*	*0,61*	*DMS-79*	*0,54*
DMS-114	Lung	CRL-2066	*NCI-H661*	*0,95*	*BT-20*	*0,67*	*SNU-423*	**−** *0,12*	*K-562*	*0,63*	*SNU-182*	*0,57*
DMS-79	Lung	CRL-2049	*SW-403*	*0,96*	*NCI-H82*	*0,68*	*SNU-449*	**−** *0,11*	*AN3-CA*	*0,63*	*SHP-77*	*0,64*
NCI-H358	Lung	CRL-5807	*C-4I*	*0,98*	*WIDR*	*0,70*	*C-33A*	**−** *0,11*	*SNU-182*	*0,66*	*MOLT-4*	*0,65*
NCI-H441	Lung	HTB-174	*DMS-114*	*0,98*	MOLT-4	0,73	*WIDR*	**−** *0,11*	*BT-20*	*0,66*	*RAJI*	*0,65*
NCI-H661	Lung	HTB-183	**RAJI**	**0,99**	**A-498**	**0,73**	*NCI-H82*	**−** *0,09*	*COLO-668*	*0,71*	*NCI-H1650*	*0,67*
NCI-H69	Lung	HTB-119	**SNU-182**	**1,01**	**HEC-1-B**	**0,73**	*SNU-182*	**−** *0,08*	**A-498**	**0,72**	**A-427**	**0,69**
NCI-H82	Lung	HTB-175	**ChaGo-K-1**	**1,01**	**SNU-423**	**0,81**	*ChaGo-K-1*	**−** *0,07*	**SK-N-AS**	**0,72**	**BT-20**	**0,69**
SHP-77	Lung	CRL-2195	**NCI-H82**	**1,02**	**NCI-H1975**	**0,82**	*RAJI*	**−** *0,05*	**RAJI**	**0,72**	**AN3-CA**	**0,71**
ChaGO-K-1	Lung	HTB-168	**MOLT-4**	**1,03**	**DMS-79**	**0,82**	*BT-20*	**−** *0,01*	**NCI-H1975**	**0,73**	**SNU-423**	**0,73**
NCI-H1650	Lung	CRL-5883	**NCI-H69**	**1,03**	**RAJI**	**0,82**	*DMS-114*	*0,00*	**ChaGo-K-1**	**0,78**	**NCI-H1975**	**0,74**
NCI-H1975	Lung	CRL-5908	**ES-2**	**1,05**	**ChaGo-K-1**	**0,83**	*HOS*	*0,01*	**SNU-423**	**0,80**	**A-498**	**0,80**
NCI-H1993	Lung	CRL-5909	**NCI-H1975**	**1,08**	**DMS-114**	**0,89**	**NCI-H1650**	**0,14**	**NCI-H661**	**0,88**	**ChaGo-K-1**	**0,81**
RD	Muscle	CCL-136	**SK-N-AS**	**1,10**	**SNU-182**	**0,90**	**RD**	**0,20**	**DMS-79**	**0,89**	**HOS**	**0,87**
CAOV3	Ovary	HTB-75	**A-427**	**1,10**	**COLO-668**	**0,91**	**SK-N-AS**	**0,20**	**SHP-77**	**0,90**	**DMS-114**	**0,93**
ES-2	Ovary	CRL-1978	**RD**	**1,12**	**HCT-8**	**0,95**	**ES-2**	**0,21**	**C-33A**	**0,91**	**SK-N-AS**	**1,04**
HOS	Sarcoma	CRL-1543	**COLO-668**	**1,13**	**A-427**	**0,98**	**HEC-1-B**	**0,31**	**DMS-114**	**0,92**	**HEC-1-B**	**1,07**
A375	Skin	CRL-1619	**DMS-79**	**1,20**	**SK-N-AS**	**1,00**	**NCI-H661**	**0,32**	**RD**	**0,96**	**RD**	**1,28**
AN3-CA	Uterus	HTB-111	**HEC-1-B**	**1,31**	**RD**	**1,01**	**A-498**	**0,49**	**HEC-1-B**	**0,98**	**COLO-668**	**1,34**
HEC-1-B	Uterus	HTB-113	**SW-620**	**1,31**	**SHP-77**	**1,04**	**AN3-CA**	**0,61**	**SW-403**	**1,08**	**SW-948**	**1,45**

For each drug, the cell lines are ranked based on their RI values (green: low, red: high, yellow: intermediate RI) at the C2 concentration. The panels of resistant- and sensitive-designated cell lines are marked by bold and the intermediate cell lines are marked by italic formatting for each agent.

### DNA Isolation and Quality Control

DNA was isolated using the Qiagen DNeasy Blood and Tissue Kit (Qiagen, Hilden, Germany, cat. no.: 69506) according to the product user’s guide. Quantity and quality of the DNA were tested by using a Nanodrop 1000 system (BCM, Houston, TX, USA). DNA (A260) and protein (A280) concentrations and sample purity (260/280 ratio) were measured and only high quality DNA was used for further analysis. DNA was stored at −80°C.

### Authentication of Cell Lines

Authentication was performed for cell lines obtained more than 4 years ago from ATCC using short tandem repeat (STR) analysis of 10 specific loci in the human genome and a mouse specific marker. Authentication was carried out by StemElite ID System at the Fragment Analysis Facility, Johns Hopkins University (Baltimore, USA). STR profiles of the applied cell lines were compared to the STR profile database of the Leibniz Institute DSMZ - German Collection of Microorganisms and Cell Cultures (http://www.dsmz.de). All cell lines included in this study were contamination-free.

### Resistance Tests

Drugs were used in their commercially available form, and were applied to the cells in 3 concentrations (C1, C2, C3). C1 = 0.1*C2 and C3 = 10*C2. Concentration C2 was deduced from the clinically used doses (see **Table 2**).

**Table 2:** Concentrations used in the cell lines to measure the resistance indexes.

Drug	Concentration (µM)		
	C1	C2	C3
lapatinib	2.867	28.67	286.7
sunitinib	0.124	1.24	12.4
sorafenib	1.147	11.47	114.7
erlotinib	0.508	5.08	50.8
gefitinib	0.745	7.45	74.5

The MTT assay (Roche, Cat. No.: 11465007001), was used to test the anti-proliferative effect of reagents and cell viability. In each experiment, 2000 cells/well were seeded in 100 µl medium onto 96-well plates. After one day incubation, precontrol cells were stained. At the same time, the cultures were treated with all 5 studied drugs at C1, C2 and C3 concentrations. On the fifth day the experiment was terminated and the cells were stained. The absorbance was read with a Thermo Scientific Multiskan® FC. The absorbance measured at 595 nm was corrected with the background measured at 690 nm. All measurements were repeated three times and for the calculation of the resistance index (RI) values, the averages of the 3 measurements were used. The resistance index (RI) was computed by the formula [26]:
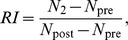
where N_pre_ is the medium absorbance value of precontrol (representing the number of cells at the beginning of the treatment), N_post_ is the medium absorbance value of control (representing the number of cells at the end of the treatment with vehicle treatment only), and N_2_ is the medium absorbance value of treated cells treated with the C2 concentration of the studied drug. C1 and C3 concentrations were used as internal controls to monitor the dynamic range of the agents. Only cell lines that fulfilled the quality criteria of N_post_>N_pre_ and deviation in cell growth within repetitions <15% were included in the evaluation.

### Feature Selection

Raw microarray data for the cell lines were generated in the GSK caArray project (ftp://caftpd.nci.nih.gov/pub/caARRAY/transcript_profiling). caArray was developed using the caBIG compatibility guidelines, as well as the Microarray Gene Expression Data (MGED) society standards for microarray data. After downloading, the raw.CEL files were MAS5 normalized in the R statistical environment (www.r-project.org) using the affy Bioconductor library [27]. MAS5 ranked among the best normalization methods when compared to the results of qRT-PCR measurements in our recent study [28]. Each cell line was measured on the microarrays by triplicates - in the final step of the pre-processing the average of these were computed. As probe sets with very low abundance are not only unlikely to hold biological significance, but are also error prone, we made a filtering to retain only probe sets with an average expression over 100 and maximal expression over 1000. The complete normalized database is presented in **Table S1**.

The complete dataset consisting of the expression profiles has been arranged into 2 classes, according to the resistance properties of the cell lines. Intermediate cell lines were excluded. This selection procedure resulted in 5 datasets, which were treated as autonomous classification tasks. To obtain the list of genes best correlated to resistance, we used Significance Analysis of Microarrays (SAM) [29] and rank products [30], [31]. While SAM is the most widely used method, rank products were found to deliver the best performance in a setting similar to our project with low sample size in an earlier summary of available feature selection methods [32]. The efficacy of the gene sets to discriminate resistant and sensitive cell lines was computed using Prediction Analysis of Microarrays [33]. The R file of the used statistical analysis is available in the supplemental material as Script S1.

To assess the capability of the gene-sets to predict survival, we searched in Pubmed GEO for datasets with available clinical follow-up where cancer patients were treated with one of the five investigated agents. Finally, to search for gene lists similar to our genes and to identify genes correlated to the identified genes, we used the CCancer search engine [34].

### RNA Isolation and Quality Control

After homogenization using Qiashredder, RNA was isolated using the Qiagen RNeasy kit (Qiagen, Hilden, Germany) according to the product user’s guide. Quantity and quality of the isolated RNA was tested by using a Nanodrop 1000 system (BCM, Houston, TX, USA) and by gel electrophoresis using an Agilent Bioanalyzer system (Agilent Technologies Inc., Santa Clara, CA, USA). RNA (A260) and protein (A280) concentrations and sample purity (260/280 ratio) were measured. Only high quality, intact total RNA was accepted for samples which also showed regular 18S and 28S ribosomal RNA bend pattern on the Bioanalyzer analysis. RNA was kept at -80°C until RT-PCR measurement.

### TaqMan Assay

TaqMan real-time PCR was used to measure the expression of 95 selected genes (plus one housekeeping gene) using a Micro Fluidic Card System (Applied Biosystems, Foster City, CA, USA) in 40 cell lines. The measurements were performed using an ABI PRISM® 7900HT Sequence Detection System as described in the products User Guide. The genes were selected to include the top genes correlated to resistance to the various agents. Additionally, based on a literature search, a set of genes correlated to targeted therapy resistance and members of the EGFR/RAS pathway were also added for additional analyses. The list of included genes is presented in **Table S2**.

### Data Analysis of the RT-PCR Measurements

For primary data analysis the SDS 2.2 software was used. The delta Ct values (which represent the expression normalized to ribosomal 18S expression) were grouped according to the resistance characteristics against the various agents into groups. Then, student’s t-test was performed to compare the expression of the gene in the various groups independently. Statistical significance was set at p<0.05.

### Renal Cell Carcinoma (RCC) Sample Collection

Patients were treated at the Department of Urology, Semmelweis University, Budapest, Hungary between 2005 and 2010. Samples were collected according to state-of-the-art pathology protocol from all patients operated for renal cell cancer. However, only patients with later metastatic disease were included in present study, as only these patients received a targeted therapy treatment. The two agents in clinical use for metastatic RCC are sunitinib and sorafenib. Of these, sunitinib is administered in the first line setting, thus, these patients were chosen for the immunohistochemical analyses. Tissue microarrays (TMA) of all FFPE samples were constructed with the Tissue Micro-Array Builder instrument (Histopathology Ltd., Pécs, Hungary). In the TMAs, duplicates of 2 mm wide cores were used of each tumor representing their most relevant areas according to histopathology.

### Immunohistochemistry

The immunohistochemical (IHC) reactions were performed on 4 µm thick sections obtained from TMA blocks. After deparaffinization, the slides were heated in a microwave oven in Target Retrieval Solution (DAKO, Carpenteria, CA, USA) for 30 minutes. An automated Ventana Benchmark immunostainer system was used according to the protocol ‘880’ (and ‘870’ for LGALS8) provided by the manufacturer (Ventana Medical Systems Inc., Tucson, AZ, USA). RAB17 (dilution, 1∶200), LGALS8 (1∶50), EpCam (1∶100) and CD9 (1∶300) antibodies were used for staining. The tissues were counterstained with Mayer’s hemalaun (00-8011, Zymed Laboratories Inc.). Positive controls and negative control tissues were applied in all IHC runs. In case of CD9, LGALS8, RAB17 cytoplasmic reaction, for EpCAM membranous staining was accepted as proper localization.

The stained slides were digitalized with a slide scanner (Mirax MIDI Scan, 3DHistech Ltd., Budapest, Hungary), and intensity of the reaction (0: negative reaction, +1: weak positivity, +2: moderate positivity, +3: strong reaction) and frequency of positively stained cells (0∶0–1%, 1∶1–5%, 2∶5–10%, 3∶10–20%, 4∶20–33%, 5∶33–50%, 6∶50–66%, 7∶66–80%, 8∶80–100%) were evaluated separately. Finally, correlation between 25-percentile survival groups as well as Kaplan-Meier survival plots based on grouping using the median were computed in WinStat for Excel (R. Fitch Software, Bad Krozingen, Germany).

## Results

### Resistance Tests

The resistance of each cell line was measured in triplicates for each of the three concentrations of the five agents. Then, the cell lines were ranked based on their RI values. An intermediate RI value was designated as being within the median RI value +/−10% of the RI range. Cell lines exhibiting higher RIs were designated as resistant, and cell lines with lower RIs as sensitive. A complete overview of the separation of cell lines into groups is depicted in **Table 1** **.**

### Identification of Discriminatory Genes

For the classification, the genes were filtered to include only those which achieved an at least 2-fold difference in the average expression compared between cell lines designated as sensitive or resistant. Then, feature selection with SAM and rank products was performed. Only those genes were accepted as significant, which achieved a false discovery rate below 20%. The complete list of significant genes is listed in **Table S3**.

The accuracy of the classification in the leave-one-out cross-validation setting using all genes in the cell lines resulted in an efficiency of 92.8% in PAM (cell lines with intermediate resistances were excluded). The use of the top 100 genes identified by rank products resulted in 79% correct predictions. The correct classifications using the top 100 rank products identified genes are presented in blue and incorrect classifications in red in **Table S4**.

Although the investigated agents are in clinical use already for over 7 years, we were unable to find published data sets suitable for meta-analysis of the identified gene-set. Thus, we could not perform an *in silico* validation on prediction of clinical response or survival. Using CCancer, all together 27 publications with overlapping gene sets have been identified. These are presented in **Table S5**.

### TaqMan Validation of Cell Line-derived Gene Profiles

TaqManq RT-PCR results are summarized in **Table 3**. 45 of the 63 genes associated with resistance in the feature selection using the microarray data were confirmed below p<0.05 and 23 of these below p<0.01. The highest significance was achieved by *ITGB4* (p = 0.005) of the erlotinib-resistance associated, by *IADA* (p = 0.003) of the gefitinib-associated genes, by *FAT4* (p = 0.011) of the sorafenib associated genes and by *FURIN* and *ME1* (p = 0.011) of the lapatinib-associated genes. Several genes were significantly confirmed of the sunitinib-resistance gene signature including *KRT18* (p = 0.001), *LGALS8* (p = 0.019), *RAB17* (p = 0.002), *CD9* (p = 0.002) and *PPL* (p = 0.001). Meanwhile, only 7 of the 32 genes previously described in the literature as associated with resistance against the targeted therapy agents were confirmed. The complete normalized result of the TaqMan assays is available as **Table S6**.

**Table 3:** Validation of the top genes by TaqMan RT-PCR in the cell lines.

Symbol	TaqMan ID	Affymetrix ID	Gene name		p value
**Erlotinib**					
B3GNT3	Hs00429537_m1	204856_at	UDP-GlcNAc:betaGal beta-1,3-N-acetylglucosaminyltransferase 3		**0.021**
CAST	Hs00156280_m1	208908_s_at	calpastatin		**0.030**
CLMN	Hs00226865_m1	221042_s_at	calmin (calponin-like, transmembrane)		**0.023**
ERBB3	Hs00176538_m1	202454_s_at	v-erb-b2 erythroblastic leukemia viral oncogene homolog 3		**0.016**
FXYD5	Hs00204319_m1	218084_x_at	FXYD domain containing ion transport regulator 5		**0.016**
ITGB4	Hs00236216_m1	204990_s_at	integrin, beta 4		**0.005**
LGALS3	Hs00173587_m1	208949_s_at	lectin, galactoside-binding, soluble, 3		**0.016**
NEAT1	Hs01008264_s1	214657_s_at	nuclear paraspeckle assembly transcript 1		**0.019**
PRSS22	Hs00223188_m1	205847_at	protease, serine, 22		**0.023**
S100A10	Hs00741221_m1	200872_at	S100 calcium binding protein A10		**0.015**
SECTM1	Hs00171088_m1	213716_s_at	secreted and transmembrane 1		**0.026**
TFAP2C	Hs00231476_m1	205286_at	transcription factor AP-2 gamma		**0.004**
**Gefitinib**					
ADA	Hs01110945_m1	216705_s_at	adenosine deaminase		**0.003**
COL5A1	Hs00609088_m1	212489_at	collagen, type V, alpha 1		**0.018**
SLC2A6	Hs01115485_m1	220091_at	solute carrier family 2, member 6		**0.027**
**Sorafenib**					
FAT4	Hs01570491_m1	219427_at	FAT tumor suppressor homolog 4		**0.011**
GNG11	Hs00914578_m1	204115_at	guanine nucleotide binding protein (G protein), gamma 11		**0.068**
TUSC3	Hs00954406_m1	213423_x_at	tumor suppressor candidate 3		**0.021**
**Sunitinib**					
CD9	Hs00233521_m1	201005_at	CD9 molecule		**0.002**
EPCAM	Hs00158980_m1	201839_s_at	epithelial cell adhesion molecule		**0.009**
KRT18	Hs01941416_g1	201596_x_at	keratin 18		**0.001**
KRT8	Hs01630795_s1	209008_x_at	keratin 8		**0.041**
LGALS8	Hs00374634_m1	208934_s_at	lectin, galactoside-binding, soluble, 8		**0.019**
LSR	Hs00210880_m1	208190_s_at	lipolysis stimulated lipoprotein receptor		**0.046**
PPL	Hs00160312_m1	203407_at	periplakin		**0.001**
RAB17	Hs00940833_m1	218931_at	RAB17, member RAS oncogene family		**0.002**
SAT1	Hs00161511_m1	203455_s_at	spermidine/spermine N1-acetyltransferase 1		**0.003**
SIGIRR	Hs00222347_m1	52940_at	single immunoglobulin and toll-interleukin 1 receptor domain		**0.004**
**Lapatinib**					
FURIN	Hs00965485_g1	201945_at	furin (paired basic amino acid cleaving enzyme)		**0.011**
ME1	Hs00159110_m1	204059_s_at	malic enzyme 1, NADP(+)-dependent, cytosolic		**0.011**
TMOD3	Hs00205710_m1	220800_s_at	tropomodulin 3		**0.004**
**Genes associated with resistance against multiple agents**					
AGR2	Hs00180702_m1	209173_at	anterior gradient homolog 2	*sunitinib*	**0.013**
ANXA3	Hs00971411_m1	209369_at	annexin A3	*gefitinib*	**0.040**
				*sorafenib*	**0.032**
				*sunitinib*	**0.001**
				*lapatinib*	**0.045**
CDH1	Hs01023894_m1	201130_s_at	cadherin 1, type 1, E-cadherin	*erlotinib*	**0.050**
				*sunitinib*	**0.002**
CLDN7	Hs00600772_m1	202790_at	claudin 7	*sunitinib*	**0.013**
COL3A1	Hs00943809_m1	215076_s_at	collagen, type III, alpha 1	*gefitinib*	**0.005**
				*sorafenib*	**0.000**
				*sunitinib*	**0.009**
FXYD3	Hs00254211_m1	202488_s_at	FXYD domain containing ion transport regulator 3	*erlotinib*	**0.037**
				*sunitinib*	**0.016**
GJA1	Hs00748445_s1	201667_at	gap junction protein, alpha 1, 43 kDa	*sunitinib*	**0.000**
KRT19	Hs00761767_s1	201650_at	keratin 19	*erlotinib*	**0.032**
				*sorafenib*	**0.029**
				*sunitinib*	**0.000**
LHX2	Hs00180351_m1	211219_s_at	LIM homeobox 2	*sorafenib*	**0.021**
MPZL2	Hs00170684_m1	203780_at	myelin protein zero-like 2	*sorafenib*	**0.036**
				*sunitinib*	**0.008**
NEFH	Hs00606024_m1	33767_at	neurofilament, heavy polypeptide	*erlotinib*	**0.001**
				*sunitinib*	**0.002**
RAB25	Hs00220628_m1	218186_at	RAB25, member RAS oncogene family	*erlotinib*	**0.006**
				*gefitinib*	**0.011**
				*sorafenib*	**0.015**
				*sunitinib*	**0.007**
S100P	Hs00195584_m1	204351_at	S100 calcium binding protein P	*sunitinib*	**0.024**
TACSTD2	Hs00242741_s1	202286_s_at	tumor-associated calcium signal transducer 2	*sunitinib*	**0.037**
**confirmed literature-based genes**					
ERBB2	Hs01001580_m1	216836_s_at	v-erb-b2 oncogene	*lapatinib*	**0.034**
TGFA	Hs00608187_m1	205016_at	transforming growth factor, alpha	*lapatinib*	**0.034**
ANGPT1	Hs00375822_m1	205608_s_at	angiopoietin 1	*sunitinib*	**0.036**
IFNG	Hs00989291_m1	210354_at	interferon, gamma	*sunitinib*	**0.024**
PDGFA	Hs00964426_m1	205463_s_at	platelet-derived growth factor alpha polypeptide	*sunitinib*	**0.044**
AKT1	Hs00178289_m1	207163_s_at	v-akt murine thymoma viral oncogene homolog 1	*erlotinib*	**0.047**
				*lapatinib*	**0.041**
COX2	Hs00153133_m1	204748_at	cyclooxygenase	*sunitinib*	**0.034**

For the complete results of RT-PCR measurements refer to Table S6.

**Figure 2:**
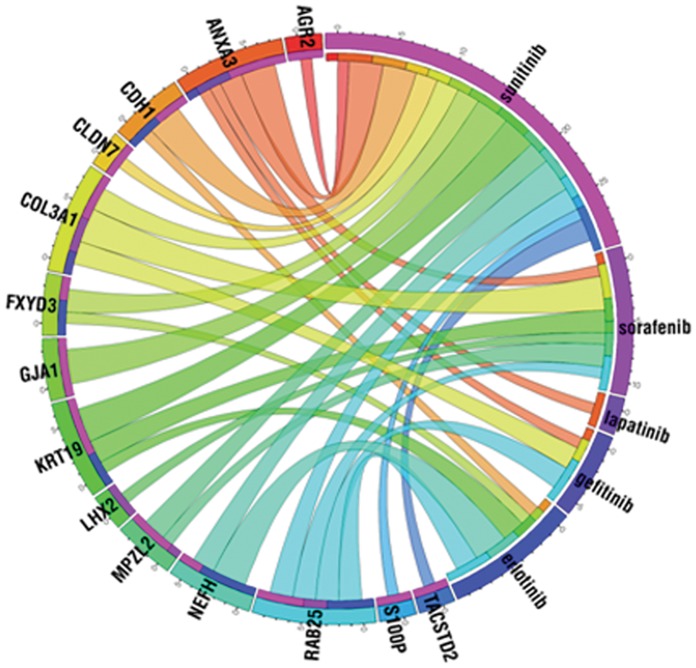
Circos plot of genes conferring multiple resistances. Circos plot of RT-PCR validated correlations for genes associated with resistance against multiple agents as identified by microarray analysis. The thickness of the ribbons correlate to the *log(p)* of the correlation (see Table 2.). Note the high number of genes associated with sunitinib resistance and the single gene associated with lapatinib resistance. The two most informative genes are ANXA3 and RAB25, each associated with resistance against four agents.

Some of the genes were associated with resistance against several agents. The highest significance of these was achieved by *COL3A1* (p<0.001 in case of sorafenib-resistance), *GJA1* (p<0.001 in case of sunitinib-resistance) and *KRT19* (p<0.001 in case of sunitinib-resistance). We have also depicted the genes associated with resistance against multiple agents using a circus-plot (see **Figure 2**). Using this approach one can recognize the high number of genes associated with sunitinib resistance and the presence of only a single gene correlated to lapatinib resistance. Only two genes (*ANXA3* and *RAB25*) were correlated to intrinsic resistance against at least four agents.

### IHC-based Validation in Renal Cell Carcinomas

**Figure 3:**
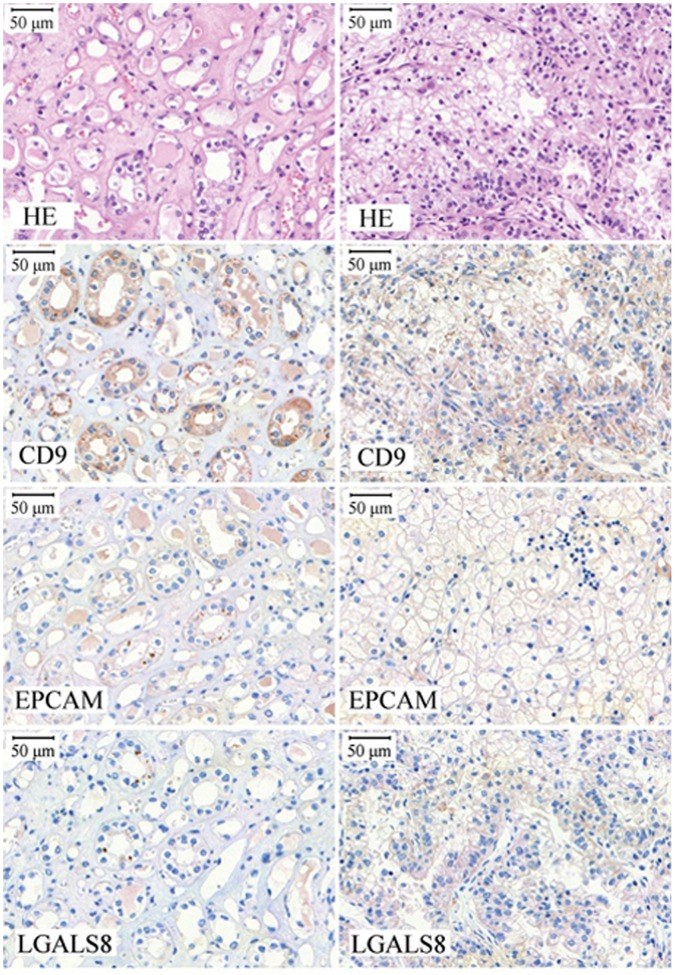
Immunohistochemistry. Representative examples of the immunhistochemical validation for CD9, EpCAM and LGALS8. Left column: normal kidney, right column: selected tumor tissue.

Altogether 39 sunitinib-treated patients with metastatic renal cell carcinoma were included in the study. The patient samples were collected before the administration of first-line TKI and are therefore similar to the measurement of gene expression in cell lines without treatment. The average age of the patients was 59 years, 63% of patients were female. The median overall survival is 14 months with 12/39 deaths. The average survival is 20 months. Partial metastasectomy was performed in case of seventeen patients. Representative images of the immunohistochemical staining for three proteins encoded by the identified genes are displayed in **Figure 3**. The detailed results in all samples for all genes are presented in **Table S7**.

**Figure 4:**
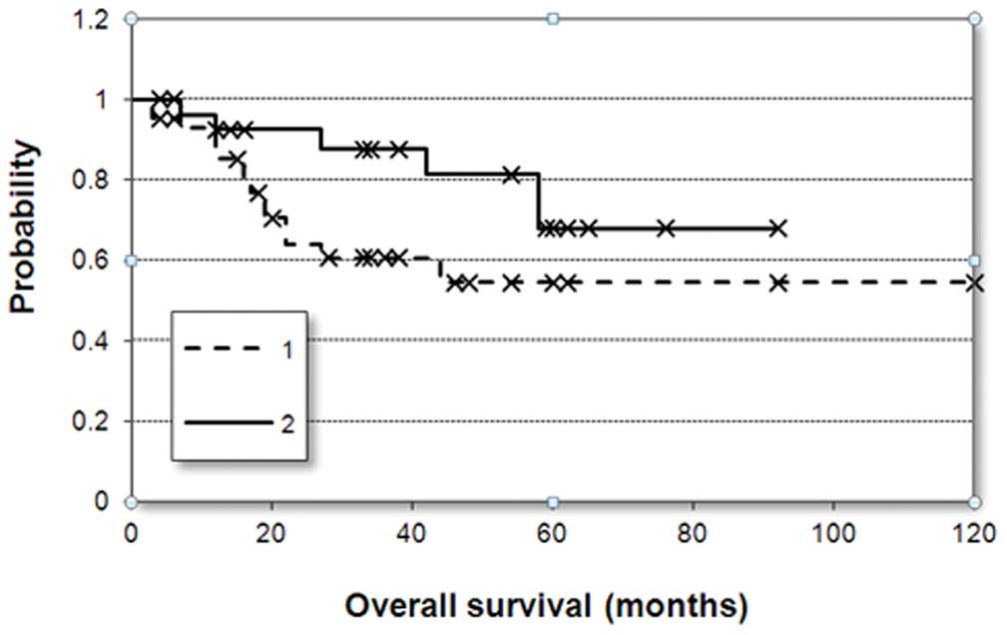
Survival plots. Kaplan-Meier survival plots of sunitinib-treated metastatic RCC samples divided into two cohorts based on the median of EpCAM positive cells (p = 0.01).

The increased staining intensity of LGALS8 (p = 0.026) and RAB17 (p = 0.018) and the frequency of positive cells for EpCAM (p = 0.01) and LGALS8 (p = 0.01) were correlated to improved survival. Meanwhile, CD9 - although showing a trend towards reduced survival in patients having increased staining intensity (p = 0.14) - was not significant. The Kaplan-Meier survival plot for EPCAM is depicted in **Figure 4**.

## Discussion

Targeted therapy agents acting via the ERBB/RAS pathway entered the mainstream cancer therapy guidelines. As still only 10–47% of patients respond to these therapies, it is of utmost importance to identify the drivers and potential markers of resistance. In our study we used 45 cancer cell lines and genome-wide gene expression signatures to identify potential new intrinsic biomarker genes. As a potential clinical application of our strategy, we validated the products of resistance-associated genes by IHC analysis in sunitinib-treated renal cell carcinomas.

We used a heterogeneous panel of cancer cell lines originating from lung (used TKIs include erlotinib and gefitinib), breast (lapatinib), renal (sorafenib and sunitinib), and liver (sorafenib). Cell lines with a known RAS mutation were excluded, since activating RAS mutations render the inhibition of upstream tyrosine kinases completely ineffective, as has been previously shown for colon cancer [35]. The selection of cell lines enables identification of robust genes related to previously unidentified independent pathways.

In a recent study of Barretina et al, a large panel of cell lines was investigated to identify markers of sensitivity against a set of cytotoxic and targeted agents including three of the tyrosine kinase inhibitors used in present study (erlotinib, lapatinib and sorafenib) by measuring sensitivity at the IC50 and EC50 values [36]. To increase clinical relevance of cancer cell line testing, we used drug concentrations applied in clinical settings, as we expected to find the most reliable candidate markers at concentrations also achievable in patients [37], [38]. The robustness of the approach using such pre-defined clinical concentrations is supported by the successful validation in a clinical cohort of sunitinib-treated patients.

We have found most cross-resistance associated genes related to sunitinib-resistance. Interestingly, so far only a few genes have been correlated with sunitinib-resistance in the literature while the number of candidate genes involved in resistance against the other agents is much larger. Therefore, we particularly focused on sunitinib resistance and performed immunohistochemical experiments on tumor samples to validate the discriminatory potential of four new candidate biomarkers, *LGALS8*, *RAB17*, *EpCAM*, and *CD9*.

Our first candidate gene *LGALS8* encodes a member of the galectin family. Galectins have been implicated in many functions including development, differentiation, cell-cell adhesion, cell-matrix interaction, growth regulation, apoptosis, and RNA splicing. Galectin-8 may also be involved in angiogenesis [39], and the expression is changed during hypolaryngeal and laryngeal tumor progression [40]. The second gene, *RAB17* is an epithelial cell-specific GTPase playing an important role in the regulation of membrane trafficking [41]. The third gene, epithelial cell adhesion molecule (*EpCAM*) is a membrane protein with proto-oncogenic properties that is expressed in numerous cancers and is a promising anticancer drug target. It functions as a homotypic calcium-independent cell adhesion molecule. The release of the intracellular domain of the molecule results in the activation of the WNT pathway [42]. High expression of *EpCAM* is associated with poor prognosis in gallbladder carcinoma [43]. Finally, *CD9* plays a role in many cellular processes including differentiation, adhesion, signal transduction, growth, and in the suppression of cancer cell motility and metastasis. Miyake *et al* demonstrated that in patients with invasive ductal carcinomas the decreased expression of *CD9* protein was associated with poor prognosis [44]. The IHC staining results confirmed the correlations between *LGALS8, RAB17* and *EpCAM* and survival of renal carcinoma patients treated with sunitinib, while CD9 failed to achieve significant discriminatory potential.

According the results of our study these genes might represent new candidates to identify patients who may benefit from sunitinib therapy. While the immunohistochemical analysis validated 3 of the 4 biomarker candidates, a larger clinical study will be needed to rigorously estimate the power and confirm their clinical significance.

In the last decade, oncogene addiction has been acknowledged as one of the key factors of cancer evolution that can also mark pathways and genes for targeted therapies [45]. However, due to the adaptation of cancer cells, drug addiction resulting from intensive treatment can overcome oncogene addiction as has been recently demonstrated in lung cancer cell lines [46]. To understand these processes, the identification of genes, which share a functional role in the resistance against several targeted therapy agents, is of high priority.

Despite the similar mechanism of action, no gene was identified to be correlated with the sensitivity against all five agents in our study. Two genes, *ANXA3* and *RAB25* were related to four drugs. *Annexin 3* (ANXA3) plays a role in cellular growth and signal transduction [47], and was previously linked to platinum resistance in ovarian cancer [48]. The product of the *ANXA3* gene was also identified as one of the tyrosine-phosphorylation targets of EGFR by immunoprecipitation and western blotting [49]. *ANXA3* was identified as one of the four down-regulated genes involved in prostate cancer progression in a recent study that compared EGFR mutated and non-mutated tumours [50]. Our results imply the possibility of the involvement of *ANXA3* in collateral pathways enabling cancer cells to circumvent TKI therapy.

*RAB25* is a member of the RAS oncogene family. Loss of *RAB25* was associated with human colorectal adenocarcinomas [51] and triple-negative breast cancer [52], but the gene has not yet been investigated in relationship to tyrosine kinase resistance. Future studies involving patients with simultaneously sequenced tyrosine kinases and RAS signaling pathway members are needed to assess its relevance in targeted therapy.

In summary, we present a comprehensive analysis pipeline for future studies of the investigated tyrosine kinase inhibitors. As a proof of principle we selected a set of genes associated with sunitinib resistance (the agent with the least published predictive biomarkers) for testing in a clinical cohort.

## Supporting Information

Table S1 **Normalized microarray data of all genes in all cell lines.**
(XLSX)

Table S2 **List of the genes selected for qRT-PCR validation.**
(XLSX)

Table S3 **Significant genes for each agent.**
(XLSX)

Table S4 **Cross validation.** The accuracy of the classification in the leave-one-out cross-validation setting using all genes in the cell lines resulted in an efficiency of 92.8% in PAM (cell lines with intermediate resistances were excluded). The use of the top 100 genes identified by rank products resulted in 79% correct predictions. The correct classifications using the top 100 rank products identified genes are presented in blue and incorrect classifications in red.
(XLSX)

Table S5 **Overlapping gene sets in other studies as identified using the ccancer algorithm.**
(XLSX)

Table S6 **The complete normalized result of the TaqMan assays.** CT values normalized to the housekeeping gene.
(XLSX)

Table S7 **Immunohistochemistry.** The intensity and frequency of the CD9, epCAM, LGALS8 and RAB17 staining, with the number of the sample and the patient ID.
(XLSX)

Script S1 **R file of the used statistical analysis.**
(PDF)
