# Supplementary material for: Identifying Resistance Mechanisms against Five Tyrosine Kinase Inhibitors Targeting the ERBB/RAS Pathway in 45 Cancer Cell Lines
Source: PLoS One. 2013 Mar 29;8(3):e59503. doi: 10.1371/journal.pone.0059503 (PMC3612034; doi:10.1371/journal.pone.0059503)
Supplement: Script S1 — R file of the used statistical analysis. (PDF) [file pone.0059503.s008.pdf]

Supplemental R scripts

Identifying Resistance Mechanisms against five Tyrosine Kinase Inhibitors Targeting the ERBB/RAS Pathway in 45 Cancer Cell Lines

Zsófia Pénzváltó, Bálint Tegze, A. Marcell Szász, Zsófia Sztupinszki, István Likó, Attila Szendrői, Reinhold Schäfer, Balázs Győrffy

Contents

#MAS5 normalization 2

SAMR classification 3

Rank-Products classification 5

#MAS5 normalization

```
#============================================================
#Input parameters:
	# folder: the folder containing the .CEL files
	# probelist: list of probesets to use in the final output:
"22277probeset.txt"
	#trans: (optional) if trans=T then the output will be
transposed
#output: mas5results.txt: normalized expression table for the 22277
probe set
#============================================================

mas5_sztup_simpleaffy = function(folder, probelist,trans=NULL){
library(affy)
library(simpleaffy)
setwd(folder)
affylista <- read.table(probelist, row.names=1)
files <- list.celfiles(path=folder,full.names=FALSE)
 results3 <- matrix(NA,dim(affylista)[1],length(files), dimnames =
list(row.names(affylista), paste("name", 1:length(files))))
for (i in 1:length(files)){
cel <-files[i]
ezaminta_gsm_hosszu <- unlist(strsplit(cel, "\\."))
ezaminta_gsm <- unlist(strsplit(ezaminta_gsm_hosszu, "\\-"))
ezaminta_gsm <- unlist(strsplit(ezaminta_gsm, "\\_"))
  rawdata <- ReadAffy(filenames=cel)
  if(rawdata@annotation=="hgu133a" |
rawdata@annotation=="hgu133plus2" | rawdata@annotation=="hgu133a2"){
  mas <- justMAS(rawdata,tgt=500,scale=TRUE)
  norm <- 2^exprs(mas)
  normadatok <- norm
  normadatok <-normadatok[rownames(affylista),]
  normadatok <- as.matrix(normadatok)
  fsztup <- function(x) mean = mean(x)
  atlag2 <- apply(normadatok, 2, fsztup)
  normadatok<- normadatok/(atlag2/1000)
  norm_bet2 <- normadatok
  mode(norm_bet2) <- "numeric"
  norm_bet2_r <- round(norm_bet2,digits=0 )
  results3[,i]<-norm_bet2_r
  colnames(results3)[i]<- ezaminta_gsm[1]
  print(i)
}
}
  results3 <- results3[,colSums(is.na(results3))<nrow(results3)]
  write.table(results3, file="mas5results.txt", sep="\t",
col.names=NA)
  if (is.null(trans)==FALSE){
  tres<-t(results3)
  write.table(tres, file="mas5results_trans.txt", sep="\t",
col.names=NA)
  }
  }
```

SAMR classification

```
#===========================================================

memory.limit(size = 2040)
setwd("c:/Users/sztup/Documents/tar")
library(samr)
t<-read.csv(file="tarter_expr45.csv", sep=";", header=T,
row.names=1)
q<-read.csv(file="sens_sam.csv", sep=";", header=F)
ql<-as.vector(q[1,], mode="numeric")
x<-t[,c(ql!=3)] #delete intermediers
y<-ql[c(ql!=3)] #delete intermediers
d=list(x=x,y=y,geneid=row.names(x),genenames=row.names(x),logged2=FA
LSE)
samr.obj <- samr(d, resp.type="Two class unpaired",nperms=1000)
delta.table<- samr.compute.delta.table(samr.obj)
sig<-samr.compute.siggenes.table(samr.obj, del=0.2, d, delta.table,
min.foldchange=0, all.genes=T, compute.localfdr=T)
write.table(sig$genes.up, file="all_up1.csv", sep=";", quote=F,
append=T)
write.table(sig$genes.lo, file="all_low1.csv", sep=";", quote=F,
append=T)

ql<-as.vector(q[2,], mode="numeric")
x<-t[,c(ql!=3)]
y<-ql[c(ql!=3)]
d=list(x=x,y=y,geneid=row.names(x),genenames=row.names(x),logged2=FA
LSE)
samr.obj <- samr(d, resp.type="Two class unpaired",nperms=1000)
delta.table<- samr.compute.delta.table(samr.obj)
sig<-samr.compute.siggenes.table(samr.obj, del=0.2, d, delta.table,
min.foldchange=0, all.genes=T, compute.localfdr=T)
write.table(sig$genes.up, file="all_up2.csv", sep=";", quote=F,
append=T)
write.table(sig$genes.lo, file="all_low2.csv", sep=";", quote=F,
append=T)

ql<-as.vector(q[3,], mode="numeric")
x<-t[,c(ql!=3)]
y<-ql[c(ql!=3)]
d=list(x=x,y=y,geneid=row.names(x),genenames=row.names(x),logged2=FA
LSE)
samr.obj <- samr(d, resp.type="Two class unpaired",nperms=1000)
delta.table<- samr.compute.delta.table(samr.obj)
sig<-samr.compute.siggenes.table(samr.obj, del=0.2, d, delta.table,
min.foldchange=0, all.genes=T, compute.localfdr=T)
write.table(sig$genes.up, file="all_up3.csv", sep=";", quote=F,
append=T)
write.table(sig$genes.lo, file="all_low3.csv", sep=";", quote=F,
append=T)

ql<-as.vector(q[4,], mode="numeric")
x<-t[,c(ql!=3)]
y<-ql[c(ql!=3)]
```

```
d=list(x=x,y=y,geneid=row.names(x),genenames=row.names(x),logged2=FA
LSE)
samr.obj <- samr(d, resp.type="Two class unpaired",nperms=1000)
delta.table<- samr.compute.delta.table(samr.obj)
sig<-samr.compute.siggenes.table(samr.obj, del=0.2, d, delta.table,
min.foldchange=0, all.genes=T, compute.localfdr=T)
write.table(sig$genes.up, file="all_up4.csv", sep=";", quote=F,
append=T)
write.table(sig$genes.lo, file="all_low4.csv", sep=";", quote=F,
append=T)

ql<-as.vector(q[5,], mode="numeric")
x<-t[,c(ql!=3)]
y<-ql[c(ql!=3)]
d=list(x=x,y=y,geneid=row.names(x),genenames=row.names(x),logged2=FA
LSE)
samr.obj <- samr(d, resp.type="Two class unpaired",nperms=1000)
delta.table<- samr.compute.delta.table(samr.obj)
sig<-samr.compute.siggenes.table(samr.obj, del=0.2, d, delta.table,
min.foldchange=0, all.genes=T, compute.localfdr=T)
write.table(sig$genes.up, file="all_up5.csv", sep=";", quote=F,
append=T)
write.table(sig$genes.lo, file="all_low5.csv", sep=";", quote=F,
append=T)
```

Rank-Products classification

```
#===========================================================

 topGenesztup2<-function (x, cutoff = NULL, method = "pfp", num.gene
= NULL,
    logged = TRUE, logbase = 2, gene.names = NULL)
{
    pfp = as.matrix(x$pfp)
    FC = as.matrix(x$AveFC)
    pval = as.matrix(x$pval)
    if (is.null(x$RPs)) {
        RP = as.matrix(x$RSs)
        rank = as.matrix(x$RSrank)
    }
    else {
        RP = as.matrix(x$RPs)
        rank = as.matrix(x$RPrank)
    }
    if (is.null(num.gene) & is.null(cutoff))
        stop("No selection criteria is input, please input either
cutoff or num.gene")
    RP.sort.upin2 = sort(RP[, 1], index.return = TRUE)
    RP.sort.downin2 = sort(RP[, 2], index.return = TRUE)
    if (!is.null(cutoff)) {
        if (method == "pfp") {
            cutgenes.upin2 = which(pfp[RP.sort.upin2$ix, 1] <
                cutoff)
            cutgenes.downin2 = which(pfp[RP.sort.downin2$ix,
                2] < cutoff)
        }
        else {
            if (method == "pval") {
                cutgenes.upin2 = which(pval[RP.sort.upin2$ix,
                  1] < cutoff)
                cutgenes.downin2 = which(pval[RP.sort.downin2$ix,
                  2] < cutoff)
            }
            else {
                stop("No criterion is input to select genes, please
select either pfp(fdr) or pval(P-value)")
            }
        }
        if (length(cutgenes.upin2) > 0) {
            numTop = max(cutgenes.upin2)
            gene.sel.upin2 = RP.sort.upin2$ix[1:numTop]
            rm(numTop)
        }
        else {
            gene.sel.upin2 = c()
        }
        if (length(cutgenes.downin2) > 0) {
            numTop = max(cutgenes.downin2)
            gene.sel.downin2 = RP.sort.downin2$ix[1:numTop]
            rm(numTop)
        }
```

```
        else {
            gene.sel.downin2 = c()
        }
    }
    if (is.null(cutoff) & !is.null(num.gene)) {
        if (num.gene > 0) {
            gene.sel.upin2 = RP.sort.upin2$ix[1:num.gene]
            gene.sel.downin2 = RP.sort.downin2$ix[1:num.gene]
        }
        else {
            gene.sel.upin2 = c()
            gene.sel.downin2 = c()
        }
    }
    if (!is.null(gene.names)) {
        if (dim(pfp)[1] != length(gene.names)) {
            cat("Warning: gene.names should have the same length as
the gene vector.",
                "\n")
            cat("No gene.names are assigned", "\n")
        }
        else {
            rownames(pfp) = gene.names
        }
    }
    pfp = round(pfp, 4)
    pval = round(pval, 4)
    RP = round(RP, 4)
    if (logged) {
        FC = round(logbase^FC, 4)
    }
    else {
        FC = round(FC, 4)
    }
if (require(hgu133plus2.db)) {
    affy.id.upin2=rownames(pfp)[gene.sel.upin2]
    symbols.upin2 <- aafSymbol(affy.id.upin2, "hgu133plus2.db")
    symbols.upin2 <-getText(symbols.upin2)

  }

    if (length(gene.sel.upin2) > 0) {

        Out.table.upin2 =
cbind(rownames(pfp)[gene.sel.upin2],symbols.upin2,gene.sel.upin2,
RP[gene.sel.upin2,
            1], FC[gene.sel.upin2], pfp[gene.sel.upin2, 1],
pval[gene.sel.upin2,
            1])


        rownames(Out.table.upin2) = rownames(pfp)[gene.sel.upin2]
        colnames(Out.table.upin2) =
c("affyid","gene.symbol","gene.index", "RP/Rsum",
            "FC:(class1/class2)", "pfp", "P.value")
```

```
        cat("Table1: Genes called significant under class1 <
class2",
            "\n\n")
    }
    else {
        cat("No genes called significant under class1 < class2",
            "\n\n")
        Out.table.upin2 = NULL
    }
if (require(hgu133plus2.db)) {
    affy.id.downin2=rownames(pfp)[gene.sel.downin2]
    symbols.downin2 <- aafSymbol(affy.id.downin2, "hgu133plus2.db")
    symbols.downin2 <-getText(symbols.downin2)

  }
    if (length(gene.sel.downin2) > 0) {
        Out.table.downin2 = cbind(rownames(pfp)[gene.sel.downin2],
symbols.downin2,gene.sel.downin2, RP[gene.sel.downin2,
            2], FC[gene.sel.downin2], pfp[gene.sel.downin2, 2],
            pval[gene.sel.downin2, 2])
        rownames(Out.table.downin2) =
rownames(pfp)[gene.sel.downin2]
        colnames(Out.table.downin2) =
c("affyid","gene.symbol","gene.index", "RP/Rsum",
            "FC:(class1/class2)", "pfp", "P.value")
        cat("Table2: Genes called significant under class1 >
class2",
            "\n\n")
    }
    else {
        cat("No genes called significant under class1 > class2",
            "\n\n")
        Out.table.downin2 = NULL
    }
    list(Table1 = Out.table.upin2, Table2 = Out.table.downin2)
}
memory.limit(size = 2040)
setwd("c:/Users/sztup/Documents/tar")
library(RankProd)
library(annaffy)
t<-read.csv(file="tarter_expr45.csv", sep=";", header=T,
row.names=1)
q<-read.csv(file="sens1.csv", sep=";", header=F)
ql<-as.vector(q[1,], mode="numeric")
t2<-t[,c(ql!=2)]
ql2<-ql[c(ql!=2)]
RP.out<-RP(t2, ql2, logged=F, rand=123)
proszt<-topGenesztup2(RP.out, num.gene=200,
gene.names=row.names(t2))
write.table(proszt, file="lista200.csv", sep=";", row.names=F,
col.names=T, quote=F, append=T)
ql<-as.vector(q[2,], mode="numeric")
t2<-t[,c(ql!=2)]
ql2<-ql[c(ql!=2)]
RP.out<-RP(t2, ql2, logged=F, rand=123)
```

```
proszt<-topGenesztup2(RP.out, num.gene=200,
gene.names=row.names(t2))
write.table(proszt, file="lista200.csv", sep=";", row.names=F,
col.names=T, quote=F, append=T)
ql<-as.vector(q[3,], mode="numeric")
t2<-t[,c(ql!=2)]
ql2<-ql[c(ql!=2)]
RP.out<-RP(t2, ql2, logged=F, rand=123)
proszt<-topGenesztup2(RP.out, num.gene=200,
gene.names=row.names(t2))
write.table(proszt, file="lista200.csv", sep=";", row.names=F,
col.names=T, quote=F, append=T)
ql<-as.vector(q[4,], mode="numeric")
t2<-t[,c(ql!=2)]
ql2<-ql[c(ql!=2)]
RP.out<-RP(t2, ql2, logged=F, rand=123)
proszt<-topGenesztup2(RP.out, num.gene=200,
gene.names=row.names(t2))
write.table(proszt, file="lista200.csv", sep=";", row.names=F,
col.names=T, quote=F, append=T)
ql<-as.vector(q[5,], mode="numeric")
t2<-t[,c(ql!=2)]
ql2<-ql[c(ql!=2)]
RP.out<-RP(t2, ql2, logged=F, rand=123)
proszt<-topGenesztup2(RP.out, num.gene=200,
gene.names=row.names(t2))
write.table(proszt, file="lista200.csv", sep=";", row.names=F,
col.names=T, quote=F, append=T)
```
